# Delivery of Cancer Care in India and Canada: Opportunities for Bidirectional Learning

**DOI:** 10.1200/JGO.19.00302

**Published:** 2019-10-28

**Authors:** Sunu Cyriac, Christopher M. Booth

**Affiliations:** ^1^ Amala Institute of Medical Sciences, Thrissur, India; ^2^ Queen’s University, Kingston, Ontario, Canada; ^3^ Queen’s Cancer Research Institute, Kingston, Ontario, Canada

## INTRODUCTION

Health systems can learn from models of care in other parts of the world. Despite substantial economic, political, demographic, and sociocultural differences, each health system has strengths and limitations. Most comparative studies of health systems describe metrics of care among countries with comparable profiles. However, valuable insights may be gained by comparing health systems that are different. Comparing “apples with oranges” may identify unconventional approaches that could benefit each system.

We write this commentary as oncologists from two different systems: India and Canada. Although we are at different stages in our careers (S.C. having just finished clinical training and C.M.B. midcareer faculty), each of us has had the opportunity to live and work in both systems. This commentary evolved from a series of conversations in which we discussed what each system could learn from the other. During these conversations, it became apparent that many important concepts are not easily measured by the usual metrics. Our goal is to highlight the unique strengths of each system that could be imported to the other. We frame this commentary around clinical narratives highlighting some important themes, which are discussed in more detail. Although these three scenarios are fictional, they are inspired by cases commonly encountered in Canada and India.

## CASE STUDY NO. 1

A 75-year-old woman from Toronto arrives at the emergency room with hemoptysis. Over the next 2 months, she undergoes investigations and is diagnosed with incurable lung cancer. With her oncologist, she discusses the benefits and risks of various treatment options. The patient wishes to balance quality of life and opts to focus on symptom management. She is unable to work but has some financial support through disability insurance, and her medical care is delivered at no personal cost. During her illness, she has access to high-quality palliative care and dies at home.

## CASE STUDY NO. 2

A 60-year-old man from rural India develops pain in the buccal mucosa and contacts a healer in his village, who proposes thermal ablation. He subsequently travels 3 hours to the nearest government hospital. After additional investigations, he undergoes surgery and radiation over the next 3 months. His total expenditure, including stay and treatment, is < 1% of the cost that would be incurred for treatment in the nearby private hospital. His family explored the private hospital but realized that the added costs would risk his children’s education and place his family in lifelong debt. Even the modest costs associated with care in the government hospital have put his family in debt.

## CASE STUDY NO. 3

A 40-year-old female executive in Mumbai visits a private hospital for abdominal pain. This center also caters to foreign nationals, who come for high-end treatment at a lower cost than that offered in their own countries. After an extensive work-up, she is diagnosed with ovarian cancer and undergoes surgery and chemotherapy. Her oncologist strongly recommends that she arrange for genetic sequencing of her tumor. Although this is an added expense, she subsequently receives “cutting edge” targeted therapy. Her care is paid for with private insurance and out of pocket.

## HEALTH SYSTEMS

Although Canada has a single-payer universal health care system, most care in India is delivered in the private sector. The government-funded system in Canada constitutes > 70% of gross health care expenditure. The remaining 30% of health expenditures (which include the costs of oral medicines and dental care) are paid for by employer schemes and/or personal out-of-pocket payments.^1,2^ In Canada, there are no “private” hospitals; all hospitals are funded by the government and provide service free of charge. Within the realm of cancer care, diagnostic investigations (including pathology and imaging), surgery, radiotherapy, and intravenous chemotherapy are provided at no cost to the patient. This coverage does not extend to all oral medicines, nor does it include all intravenous medicines, which must first be approved by a program in health technology assessment (HTA). Conversely, in India, approximately 70% of health care delivery is in the private sector, where payment is predominantly out of pocket. Cancer care in the government system in India offers most services (with the exception of certain drugs and consumables) free of charge. Recently, a major program in India (Ayushman Bharat National Health Protection Mission) has taken an important step toward universal health care, although substantial challenges will need to be considered in its implementation.^3^

In Canada, physician income comes from government sources, which can involve either fee-for-service or salary models. In India, government physicians are paid fixed salaries, whereas physicians working in the private sector are paid either fixed salaries that are based on “value” to the hospital or on a per-patient basis. Although government physicians in some states are officially not permitted to have parallel private practices, it is not uncommon for them to do so. Rules regarding appropriate physician-industry relationships are clearly defined in Canada and India; however, monitoring and implementation is suboptimal in the latter.

The Canadian health system is almost exclusively based on allopathy, although some patients do explore complimentary medicine. In India, although allopathy is still the primary treatment option, alternative modalities (ie, Ayurveda, homeopathy, Siddha) are practiced widely in rural villages, where some of these services are publicly funded.

Cancer care in Canada is largely based on practice guidelines, with centralized quality monitoring and reporting. This ensures a minimum standard of care. In India, although organizations such as the Indian Council of Medical Research and the National Cancer Grid have evidence-based management guidelines, implementation is often not regulated. Hence, care becomes more “eminence based” or “experience based,” rather than “protocol based,” leading to considerable variations in practice. The choice of provider in India is therefore strongly associated with quality of care. Within the same city, a patient could receive world-class care at one institution or low-quality care at another.

Primary prevention and early diagnosis also differ substantially. Preventive strategies such as smoking cessation and vaccinations are common in Canada. Canada has a robust public health system with great emphasis on early diagnosis; this is likely the primary factor leading to the different mortality-incidence ratios in the two countries. A potential downside of greater access to care is the risk of overdiagnosis and overtreatment. This is common in high-income countries and may become a problem in some urban populations of India.

Documentation and auditing of clinical performance is standard practice in Canada. The downside is that Canadian oncologists spend a considerable amount of time with administrative matters that take them away from clinical care. There is a movement toward improved health records and documentation in India, but it is still not widely adopted.

## PATIENT PERSPECTIVES

Patients in Canada play an active role in clinical decision making; many of them gather information from their physician, patient support groups, and the Internet before making a treatment decision. Patients may opt out of treatment because of concerns about adverse effects and quality of life; however, they rarely decline treatment because of financial concerns. Most hospitals are also supported with a strong paramedical team of pharmacists, dieticians, physiotherapists, spiritual care providers, and social workers.

In India, a patient’s faith in the treating physician is the predominant determining factor in treatment choice. By necessity, the physician often also plays the role of pharmacist, social worker, and physiotherapist. Although the patient gets holistic care from one provider, the downside is the lack of time for important discussions regarding the risks/benefits of therapy, the goals of care, and prognosis.

In Canada, family members and friends play a supporting role for decision making; their role is much greater in India. At most hospitals in India, major treatments will not be performed without the presence of a caregiver. In Canada, it is not uncommon for a patient to be unaccompanied to clinic visits and even during major treatments. In India, it is not uncommon that accompanying family members request the treating doctor not to reveal the diagnosis or prognosis of the disease to the patient.

In Canada, drug regulatory approval is a separate process from drug reimbursement decisions, which are based on HTA and cost effectiveness. Accordingly, it is not uncommon for drugs to be approved by regulators but not funded by the public health care system. Until recently, HTA has been virtually nonexistent in India^4,5^; once a drug is approved by the regulator, it is available for use. The most significant concern regarding access to medicine in India is the impact on family finances. Out-of-pocket health expenditures are a major cause of financial morbidity for Indian families, whereas catastrophic financial toxicity in Canada is not common. In India, 5% to 7% of families are pushed below the poverty line each year because of health expenditures; this is even more common among families from lower economic backgrounds.^6^ The recent initiative by the National Cancer Grid to create resource-stratified evidence-based guidelines for cancer is expected to reduce catastrophic health care expenditure by emphasizing value-based care, especially because these are being linked to reimbursements under the Ayushman Bharat scheme.

Treatment delays in India are predominantly caused by the patient not seeking care until the disease becomes advanced. In Canada, treatment delays are not uncommon at the institutional level (ie, waiting 1 to 2 months for imaging or surgery). Care in Canada can be fragmented. A patient with newly diagnosed rectal cancer may travel to the cancer center on separate days over many weeks for appointments with medical, radiation and surgical oncology, central line placement, imaging, and treatment. In India, this happens in a span of a few hours to days.

Clinical research is yet to realize its full potential in India because of a relative scarcity of trained research teams and because of the perception among the general public that medical research implies patients being experimental guinea pigs. The hierarchic structure of many Indian academic medical centers can also stifle research momentum among junior faculty. Many Canadian patients access clinical trials as a component of their medical care; this is still uncommon in India.

## HEALTH CARE PROVIDERS’ PERSPECTIVE

The most obvious difference for Canadian and Indian physicians is the marked discordance in clinical volumes. Patients have more time to interact with physicians in Canada. A Canadian oncologist may see 15 to 20 patients per day, but an Indian oncologist may see 70 to 100 patients (Table 1).^7-9^ Space and infrastructure constraints lead to crowded clinics and wards in India. Because of the remarkable clinical load, physicians in India are exposed to an amazing breadth of pathology, which enhances their clinical acumen. In Canada, documentation burdens can reduce the satisfaction of practicing clinical medicine.

**TABLE 1:**
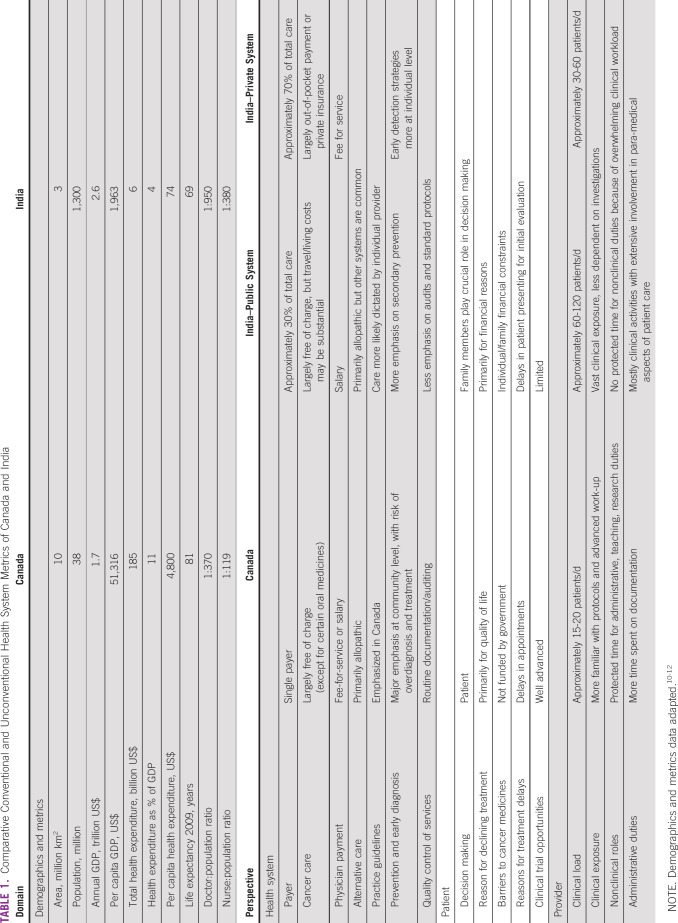
Comparative Conventional and Unconventional Health System Metrics of Canada and India

Many Canadian physicians have some protected time for nonclinical work (ie, research/education, administration). Although many leading academic centers in India emphasize research, because of the overwhelming clinical volumes, dedicated time for scholarly activities is exceedingly rare.

Finally, in Canada, a physician is still one of the most highly paid and respected professionals. In India, there is demoralization within the profession, both in terms of social dignity and payment. This has also been manifested by a recent increase in physical assaults on physicians. The reasons for this complex problem are beyond the scope of this essay; however, challenges in doctor-patient communication and unrealistic expectations from patients and families may be responsible factors.

In this commentary, we have described important differences between the cancer care systems in Canada and India. Many of these elements of system performance and clinical care are not measured in the usual health system comparative metrics. Both systems have relative strengths that could be emulated by the other. The strengths of the Canadian system include a strong public health system, reasonable clinical volumes that allow for more time with patients, protection of time for nonclinical duties, and a culture that promotes quality improvement/audit and clinical research. The strengths of the Indian system include the incorporation of many elements of diagnosis and treatment in a single patient visit, minimal wait times, and less reliance on investigations that will not alter management. Through bidirectional learning, we hope that elements of each system may be adapted to the unique circumstances of the other as we collectively seek to deliver high-quality cancer care to all patients globally.

## Data Availability

The following represents disclosure information provided by authors of this manuscript. All relationships are considered compensated unless otherwise noted. Relationships are self-held unless noted. I = Immediate Family Member, Inst = My Institution. Relationships may not relate to the subject matter of this manuscript. For more information about ASCO's conflict of interest policy, please refer to www.asco.org/rwc or ascopubs.org/jgo/site/misc/authors.html. Open Payments is a public database containing information reported by companies about payments made to US-licensed physicians (Open Payments). No potential conflicts of interest were reported.
